# Supplementary material for: Inheritance and Molecular Characterization of a Novel Mutated AHAS Gene Responsible for the Resistance of AHAS-Inhibiting Herbicides in Rapeseed (Brassica napus L.)
Source: Int J Mol Sci. 2020 Feb 17;21(4):1345. doi: 10.3390/ijms21041345 (PMC7072869; doi:10.3390/ijms21041345)
Supplement: Supplementary file 1 [file ijms-21-01345-s001.zip › Figure S1.pdf]

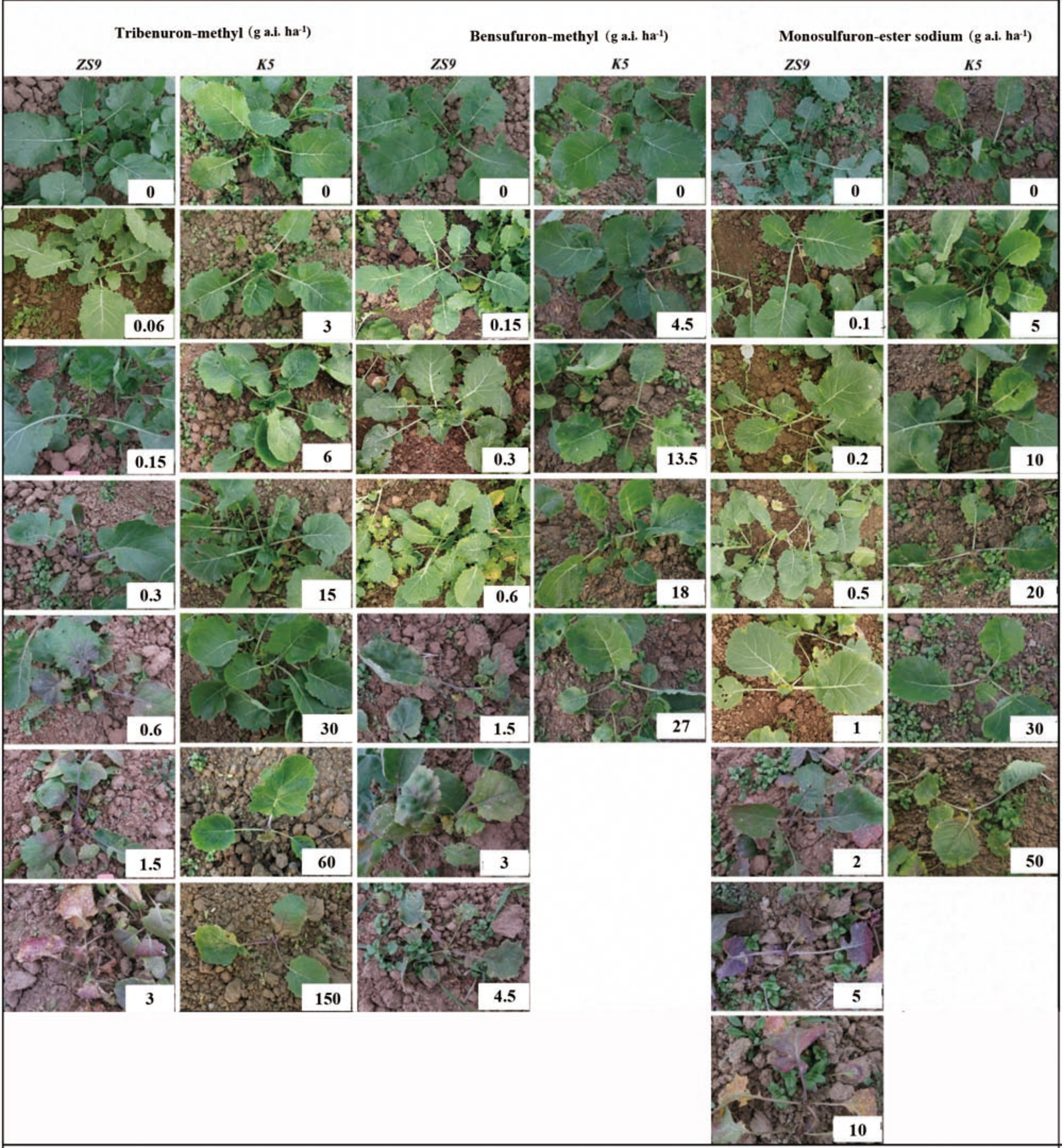

Tribenuron-methyl (g a.i. ha-1)
Bensufuron-methyl (g a.i. ha-1)
Monosulfuron-ester sodium (g a.i. ha-1)
ZS9
K5
ZS9
K5
ZS9
K5
0
0
0
0
0
0
0.06
3
0.15
4.5
0.1
5
0.15
6
0.3
13.5
0.2
10
0.3
15
0.6
18
0.5
20
0.6
30
1.5
27
1
30
1.5
60
3
2
50
3
150
4.5
5
10
