# Supplementary material for: Inheritance and Molecular Characterization of a Novel Mutated AHAS Gene Responsible for the Resistance of AHAS-Inhibiting Herbicides in Rapeseed (Brassica napus L.)
Source: Int J Mol Sci. 2020 Feb 17;21(4):1345. doi: 10.3390/ijms21041345 (PMC7072869; doi:10.3390/ijms21041345)
Supplement: Supplementary file 1 [file ijms-21-01345-s001.zip › Table S1.docx]

| Lines | Herbicides | Index | DF_1_ | MS_1_ (between groups) | MS_2_ (within groups) | F | P |
| --- | --- | --- | --- | --- | --- | --- | --- |
| ZS9 | TBM | phytotoxicity index | 6 | 0.277 | 0 | 1018.571 | 0 |
|  |  | leaf angle | 6 | 852.180 | 4.638 | 183.723 | 0 |
|  |  | leaf number | 6 | 8.913 | 0.086 | 103.264 | 0 |
|  |  | fresh weight | 6 | 270.933 | 8.505 | 31.856 | 0 |
|  |  | dry weight | 6 | 3.146 | 0.166 | 18.955 | 0 |
| K5 | TBM | phytotoxicity index | 6 | 0.172 | 0 | 360.725 | 0 |
|  |  | leaf angle | 6 | 266.500 | 27.980 | 9.525 | 0 |
|  |  | leaf number | 6 | 5.284 | 0.143 | 36.986 | 0 |
|  |  | fresh weight | 6 | 36.714 | 7.323 | 5.014 | 0.002 |
|  |  | dry weight | 6 | 0.397 | 0.116 | 3.433 | 0.016 |
| ZS9 | BSM | phytotoxicity index | 6 | 0.196 | 0 | 531.86 | 0 |
|  |  | leaf angle | 6 | 402.381 | 14.906 | 26.995 | 0 |
|  |  | leaf number | 6 | 5.603 | 0.247 | 22.683 | 0 |
|  |  | fresh weight | 6 | 136.861 | 11.518 | 11.882 | 0 |
|  |  | dry weight | 6 | 1.390 | 0.153 | 9.113 | 0 |
| K5 | BSM | phytotoxicity index | 4 | 0.072 | 0.001 | 94.548 | 0 |
|  |  | leaf angle | 4 | 12.846 | 19.093 | 0.673 | 0.626 |
|  |  | leaf number | 4 | 0.879 | 0.379 | 2.319 | 0.128 |
|  |  | fresh weight | 4 | 20.886 | 11.528 | 1.812 | 0.179 |
|  |  | dry weight | 4 | 0.128 | 0.103 | 1.237 | 0.340 |
| ZS9 | MES | phytotoxicity index | 7 | 0.354 | 0 | 1341.475 | 0 |
|  |  | leaf angle | 7 | 1203.921 | 19.049 | 63.202 | 0 |
|  |  | leaf number | 7 | 6.113 | 0.169 | 36.114 | 0 |
|  |  | fresh weight | 7 | 205.682 | 5.965 | 34.481 | 0 |
|  |  | dry weight | 7 | 2.562 | 0.095 | 27.077 | 0 |
| K5 | MES | phytotoxicity index | 5 | 0.159 | 0 | 413.747 | 0 |
|  |  | leaf angle | 5 | 304.287 | 23.822 | 12.773 | 0 |
|  |  | leaf number | 5 | 9.395 | 0.049 | 193.271 | 0 |
|  |  | fresh weight | 5 | 49.111 | 4.045 | 12.142 | 0 |
|  |  | dry weight | 5 | 0.577 | 0.060 | 9.564 | 0 |

**Table S1** Analysis of variance of five indexes of line *K5* and *ZS9*

TBM, tribenuron-methyl; BSM, bensufuron-methyl; MES, monosulfuron-ester sodium; No., number; IR, inhibition rate. *ZS9*, *Zhongshuang No*.9; *K5*, the mutant line.
