# Supplementary material for: Inheritance and Molecular Characterization of a Novel Mutated AHAS Gene Responsible for the Resistance of AHAS-Inhibiting Herbicides in Rapeseed (Brassica napus L.)
Source: Int J Mol Sci. 2020 Feb 17;21(4):1345. doi: 10.3390/ijms21041345 (PMC7072869; doi:10.3390/ijms21041345)
Supplement: Supplementary file 1 [file ijms-21-01345-s001.zip › Table S2.docx]

**Table S2** Effects of foliar-spraying of three different herbicides on morphological traits of the lines *ZS9* and *K5*

| Herbicides | Lines | Herbicide rates  (g a.i. ha^-1^ ) | Phytotoxicity Index | Leaf Angle | | Leaf number | | Fresh weight | | Dry weight | |
| --- | --- | --- | --- | --- | --- | --- | --- | --- | --- | --- | --- |
|  |  |  |  | Angle (°) | IR (%) | No. | IR (%) | Weight (g) | IR (%) | Weight (g) | IR (%) |
| TBM | *ZS9* | 0 | 0.07 + 0.02^e^ | 57.5±1.0^a^ | 0 | 6.3 ± 0.3^b^ | 0 | 20.4 ± 5.0^ab^ | 0 | 2.4 ± 0.8^a^ | 0 |
|  |  | 0.06 | 0.06 + 0.01^e^ | 58.2 ± 4.4^a^ | 0 | 7.1 ± 0.1^a^ | 0 | 23.2 ± 1.5^a^ | 0 | 2.7 ± 0.2^a^ | 0 |
|  |  | 0.15 | 0.28 + 0.01^d^ | 46.0 ± 1.1^b^ | 20.0 | 5.2 ± 0.1^c^ | 20.0 | 14.4 ± 4.1^c^ | 30.0 | 1.6 ± 0.5^b^ | 30.0 |
|  |  | 0.3 | 0.50 + 0.03^c^ | 48.5 ± 1.4^b^ | 20.0 | 5.1 ± 0.6^c^ | 20.0 | 16.4 ± 2.8^bc^ | 20.0 | 1.8 ± 0.3^b^ | 30.0 |
|  |  | 0.6 | 0.61 + 0.01^b^ | 32.5 ± 1.0^c^ | 40.0 | 3.7 ± 0.3^d^ | 40.0 | 6.0 ± 1.7^d^ | 70.0 | 0.8 ± 0.2^c^ | 70.0 |
|  |  | 1.5 | 0.64 + 0.02^b^ | 31.1 ± 2.4^c^ | 50.0 | 2.8 ± 0.3^e^ | 60.0 | 4.2 ± 1.7^d^ | 80.0 | 0.6 ± 0.2^c^ | 80.0 |
|  |  | 3 | 0.86 + 0^a^ | 11.2 ± 1.5^d^ | 80.0 | 2.5 ± 0 ^e^ | 60.0 | 2.5 ± 1.4^d^ | 90.0 | 0.4 ± 0.2^c^ | 80.0 |
|  | *K5* | 0 | 0.02 + 0.01^e^ | 47.4 ± 2.5^a^ | 0 | 6.8 ± 0.3^a^ | 0 | 15.3 ± 2.8^a^ | 0 | 1.8 ± 0.3^a^ | 0 |
|  |  | 3 | 0.11 + 0.01^d^ | 44.1 ± 6.8^ab^ | 10.0 | 7.2 ± 0.4^a^ | 0 | 14.7 ± 1.1^ab^ | 0 | 1.8 ± 0.5^a^ | 0 |
|  |  | 6 | 0.09 + 0.02^d^ | 37.5 ± 8.5^a^ | 20.0 | 6.8 ± 0.7^a^ | 0 | 13.9 ± 4.4^abc^ | 10.0 | 1.7 ± 0.3^a^ | 10.0 |
|  |  | 15 | 0.32 + 0.02^c^ | 39.0 ±1.0^a^ | 20.0 | 5.8 ± 0.4^b^ | 10.0 | 11.3 ± 1.7^abc^ | 30.0 | 1.5 ± 0.2^a^ | 20.0 |
|  |  | 30 | 0.45 + 0.02^b^ | 33.4 ± 10.8^ab^ | 30.0 | 4.8 ± 0.3^c^ | 30.0 | 10.6 ± 3.9^bcd^ | 30.0 | 1.4 ± 0.5^ab^ | 20.0 |
|  |  | 60 | 0.49 + 0.05^b^ | 34.9 ± 3.2^a^ | 30.0 | 4.3 ± 0.3^cd^ | 40.0 | 9.7 ±1.6^cd^ | 40.0 | 1.3 ± 0.2^ab^ | 30.0 |
|  |  | 150 | 0.65 + 0.05^a^ | 18.1 ± 1.7^b^ | 60.0 | 3.8 ± 0.1^d^ | 40.0 | 6.9 ± 1.7^d^ | 60.0 | 0.9 ± 0.2^c^ | 50.0 |
| BSM | *ZS9* | 0 | 0.04 + 0.01^e^ | 49.9 ± 2.5^a^ | 0 | 6.2 ± 0.6^a^ | 0 | 22.3 ± 7.0^a^ | 0 | 2.3 ± 0.8^a^ | 0 |
|  |  | 0.15 | 0.09 + 0.03^d^ | 44.4 ± 6.2^ab^ | 10.0 | 5.8 ± 0.3^a^ | 10.0 | 12.8 ± 1.2^b^ | 40.0 | 1.6 ± 0.3^b^ | 30.0 |
|  |  | 0.3 | 0.20 + 0.01^c^ | 37.9 ± 2.9^bc^ | 20.0 | 5.6 ± 0.4^a^ | 10.0 | 12.0 ± 1.1^b^ | 50.0 | 1.5 ± 0.3^b^ | 30.0 |
|  |  | 0.6 | 0.27 + 0.01^b^ | 40.6 ± 4.3^b^ | 20.0 | 5.7 ± 0.9^a^ | 10.0 | 11.4 ± 1.7^b^ | 50.0 | 1.4 ± 0.2^b^ | 40.0 |
|  |  | 1.5 | 0.62 + 0.02^a^ | 23.4 ± 1.5^d^ | 50.0 | 2.9 ± 0.5^b^ | 50.0 | 4.5 ± 2.5^c^ | 80.0 | 0.6 ± 0.3^c^ | 70.0 |
|  |  | 3 | 0.59 + 0.02^a^ | 33.5 ± 5.0^c^ | 30.0 | 3.6 ± 0.4^b^ | 30.0 | 8.4 ± 3.8^bc^ | 60.0 | 1.1 ± 0.4^c^ | 50.0 |
|  |  | 4.5 | 0.61 + 0.01^a^ | 17.1 ± 2.5^d^ | 70.0 | 2.8 ± 0.4^b^ | 60.0 | 6.0 ± 2.5^c^ | 70.0 | 0.7 ± 0.2^c^ | 70.0 |
|  | *K5* | 0 | 0.02 + 0.01^c^ | 47.4 ± 2.5^a^ | 0.0 | 6.8 ± 0.3^a^ | 0 | 1.5 ± 0.4^a^ | 0 | 1.5 ± 0.4^a^ | 0 |
|  |  | 4.5 | 0.09 + 0.03^c^ | 47.3 ± 3.5^a^ | 0.0 | 6.4 ± 0.6^a^ | 10.0 | 1.6 ± 0.4^a^ | 0 | 1.6 ± 0.4^a^ | 0 |
|  |  | 13.5 | 0.30 + 0.02^b^ | 46.1 ± 4.1^a^ | 0.0 | 5.3 ± 0.6^a^ | 20.0 | 1.3 ± 0.2^a^ | 10.0 | 1.3 ± 0.2^a^ | 10.0 |
|  |  | 18 | 0.29 + 0.04^b^ | 48.5 ± 5.7^a^ | 0.0 | 5.9 ± 0.6^a^ | 10.0 | 1.2 ± 0.4^a^ | 20.0 | 1.2 ± 0.4^a^ | 20.0 |
|  |  | 27 | 0.38 + 0.03^a^ | 43.1 ± 5.3^a^ | 10.0 | 5.9 ± 0.9^a^ | 10.0 | 1.2 ± 0.1^b^ | 20.0 | 1.2 ± 0.1^a^ | 20.0 |
| MES | *ZS9* | 0 | 0.08 + 0.02^fg^ | 61.9 ± 3.3^a^ | 0.0 | 5.3 ± 0.3^a^ | 0 | 18.2 ± 5.6^a^ | 0 | 2.2 ± 0.7^a^ | 0 |
|  |  | 0.1 | 0.06 + 0.01^h^ | 61.0 ± 2.5^ab^ | 0.0 | 5.5 ± 0.3^a^ | 0 | 18.2 ± 0.9^a^ | 0 | 2.2 ± 0.3^a^ | 10.0 |
|  |  | 0.2 | 0.10 + 0.02^f^ | 59.9 ± 6.7^ab^ | 0.0 | 4.8± 0.7^a^ | 10.0 | 13.6 ± 2.7^b^ | 30.0 | 1.6 ± 0.2^b^ | 30.0 |
|  |  | 0.5 | 0.24 + 0.01^e^ | 53.5 ± 2.1^b^ | 10.0 | 4.8 ± 0.3^a^ | 10.0 | 12.1 ± 2.4^b^ | 30.0 | 1.4 ± 0.1^b^ | 40.0 |
|  |  | 1 | 0.68 + 0.01^d^ | 30.6 ± 7.6^c^ | 50.0 | 2.4 ± 0.4^b^ | 50.0 | 3.1 ± 1.2^c^ | 80.0 | 0.5 ± 0.2^c^ | 80.0 |
|  |  | 2 | 0.74 + 0.01^c^ | 19.9 ± 2.5^d^ | 70.0 | 2.6 ± 0.6^b^ | 50.0 | 3.2 ± 0.8^c^ | 80.0 | 0.5 ± 0.1^c^ | 80.0 |
|  |  | 5 | 0.77 + 0.01^b^ | 18.5 ± 1.4^d^ | 70.0 | 2.5 ± 0.4^b^ | 50.0 | 2.3 ± 0.7^c^ | 90.0 | 0.4 ± 0.1^c^ | 80.0 |
|  |  | 10 | 0.80 + 0.02^a^ | 21.0 ± 4.4^d^ | 70.0 | 2.3 ± 0.3^b^ | 60.0 | 2.1 ± 0.3^c^ | 90.0 | 0.4 ± 0.1^c^ | 80.0 |
|  | *K5* | 0 | 0.02 + 0.02^e^ | 47.4 ± 2.5^a^ | 0 | 6.8 ± 0.3^a^ | 0 | 15.1 ± 0.5^a^ | 0 | 1.6 ± 0.1^a^ | 0 |
|  |  | 5 | 0.18 + 0.01^d^ | 48.3 ± 3.2^a^ | 0 | 5.7 ± 0.3^b^ | 20.0 | 13.9 ± 1.3^a^ | 10.0 | 1.6 ± 0.3^a^ | 0 |
|  |  | 10 | 0.20 + 0.02^d^ | 34.9 ± 2.0^b^ | 30.0 | 5.8 ± 0.1^b^ | 10.0 | 12.6 ± 1.3^ab^ | 20.0 | 1.5 ± 0.1^ab^ | 10.0 |
|  |  | 20 | 0.50 + 0.01^c^ | 36.7 ± 3.0^b^ | 20.0 | 3.1 ± 0.1^c^ | 50.0 | 10.0 ± 2.5^bc^ | 30.0 | 1.1 ± 0.3^bc^ | 30.0 |
|  |  | 30 | 0.46 + 0.02^b^ | 30.0 ± 10.1^bc^ | 40.0 | 3.1 ± 0.3^c^ | 50.0 | 7.8 ± 2.6^cd^ | 50.0 | 0.9 ± 0.3^cd^ | 40.0 |
|  |  | 50 | 0.63 + 0.03^a^ | 22.3 ± 3.3^c^ | 50.0 | 2.6 ± 0.1^d^ | 60.0 | 5.3 ± 2.0^d^ | 60.0 | 0.7 ± 0.3^d^ | 60.0 |

Phytotoxicity was scored according to seven grading standards 21 days after spraying herbicides. 0, all leaves are green; 1, young leaves (the first and second ones) are light yellow-green; 2, Partial of young leaves are yellow; 3, the second leaf is yellow and curled; 4, the mature leaves are yellow-green or light purple; 5, some mature leaves dead; 6, the plant dead. Phytotoxiciy index is calculated by the following formula:

Phytotoxicity index =$\sum\frac{\text{score of the standard × No. of plants for the }\text{correspongding}\text{ standard}}{\text{total number of plants × 7}}$

Data are expressed by mean ± standard derivation, three replications, and inhibition rate(IR) are calculated by the formula:

IR=$(1-\frac{\text{data of treatments}}{\text{data of control}} )\times100\%$

TBM, tribenuron-methyl; BSM, bensufuron-methyl; MES, monosulfuron-ester sodium; No., number. Data followed by different lower case letters within the same column indicated a significant difference at 0.05 level.
