# Supplementary material for: Inheritance and Molecular Characterization of a Novel Mutated AHAS Gene Responsible for the Resistance of AHAS-Inhibiting Herbicides in Rapeseed (Brassica napus L.)
Source: Int J Mol Sci. 2020 Feb 17;21(4):1345. doi: 10.3390/ijms21041345 (PMC7072869; doi:10.3390/ijms21041345)
Supplement: Supplementary file 1 [file ijms-21-01345-s001.zip › Table S3.docx]

**Table S3** Primers used in the present study

| Primer name | Sequence (5’- 3’) | Purpose |
| --- | --- | --- |
| *BnAHAS1*-F[29] | TCAAGAACAGTTAGATCCAC | *BnAHAS1* amplification |
| *BnAHAS1*-R[29] | GATCACCAGCTTCATCTCT |  |
| *BnAHAS2*-F[29] | AAGCAATTTCTCGCAACACTC | *BnAHAS2* amplification |
| *BnAHAS2*-R[29] | CAGAAGAGAGCATAGAATAATCAA |  |
| *BnAHAS3*-F[29] | CTCTCTCTCTCTCATCTAACCAT | *BnAHAS3* amplification |
| *BnAHAS3*-R[29] | ACTGAAACTAAGTCTTTTACCAT |  |
| *BnA1*-F1 | TGACAAAAAACGAGATTAGATTCG | Allele-specific primer |
| *BnA1*-R4 | GTTATGTTTCGTAATAGACCTCG |  |
| *ahas1*NcoI-F | CATGCCATGGATGGCGGCGGCAACATCGTCTTCTC | *Arabidopsis* transformation |
| *ahas1*NcoI-R | GCTTGGTGGATCTCCATAGAAGCACCTCCGGG |  |
| *ahas1*Blunt-F | CCCGGAGGTGCTTCTATGGAGATCCACCAAGC |  |
| *ahas1*Blunt-R | TCAGTACTTAGTGCGACCATCCCCTTC |  |
| TA1-F | TTGTAACGATGAGTTGTCCCTG | Semi-quantitative PCR primers |
| TA1-R | AACACCAAACGCCAGCAA |  |
| UBC_qPCR-F | CTGCGACTCAGGGAATCTTCTAA |  |
| UBC_qPCR-R | TTGTGCCATTGAATTGAACCC |  |

[29] Hu et al. 2012. Sci Agric Sin
