# Supplementary material for: Inheritance and Molecular Characterization of a Novel Mutated AHAS Gene Responsible for the Resistance of AHAS-Inhibiting Herbicides in Rapeseed (Brassica napus L.)
Source: Int J Mol Sci. 2020 Feb 17;21(4):1345. doi: 10.3390/ijms21041345 (PMC7072869; doi:10.3390/ijms21041345)
Supplement: Supplementary file 1 [file ijms-21-01345-s001.zip › Table S4.docx]

| No. | Code | Origin | No. | Code | Origin |
| --- | --- | --- | --- | --- | --- |
| 1 | 6C251 | *Q10C* | 16 | 6C10 | *Zhong 7* |
| 2 | 6C239 | *S11R* | 17 | 6C15 | *Zhong 5* |
| 3 | 6C172 | *S8R* | 18 | 6C21 | *Zhong 9* |
| 4 | 6C184 | *QY211R* | 19 | 6C47 | *Zhong9_III* |
| 5 | 6C216 | *HYZ01R* | 20 | 6C83 | *ZY72* |
| 6 | 6C163 | *9722* | 21 | 6C96 | *2010B1* |
| 7 | 6C206 | *SH11* | 22 | 6C70 | *H15* |
| 8 | 6C260 | *D1526* | 23 | 6C76 | *New B1* |
| 9 | 6C229 | *Z6C* | 24 | 6C109 | *2010B7* |
| 10 | 6C242 | *Q7C* | 25 | 6C124 | *2012B1* |
| 11 | 6C246 | *Q7C* | 26 | 6C100 | *2010B4* |
| 12 | 6C212 | *Y6* | 27 | 6C05 | *Zhong 2* |
| 13 | 6C214 | *2000-5R* | 28 | 6C58 | *Zhong 4* |
| 14 | 6C226 | *Z821R* | 29 | 6C64 | *CZ49* |
| 15 | 6C230 | *QSC* | 30 | 6C88 | *ZY18* |

**Table S4** Rapeseed (*Brassica napus* L.) accessions used for *AHAS* sequence conformation and verification of the AS-PCR markers
