# Supplementary material for: Inheritance and Molecular Characterization of a Novel Mutated AHAS Gene Responsible for the Resistance of AHAS-Inhibiting Herbicides in Rapeseed (Brassica napus L.)
Source: Int J Mol Sci. 2020 Feb 17;21(4):1345. doi: 10.3390/ijms21041345 (PMC7072869; doi:10.3390/ijms21041345)
Supplement: Supplementary file 1 [file ijms-21-01345-s001.zip › Table S5.docx]

| Populations | Resistant plants | Sensitive  plants | Expected  ratio | Ratio | χ^2^c | P value |
| --- | --- | --- | --- | --- | --- | --- |
| ***K5* × *ZS9*** | |  |  |  |  |  |
| F_2_ | 89 | 31 | 3:1 | 2.9:1 | 0.01 | 0.99 |
| BC_1_ | 88 | 73 | 1:1 | 1.2:1 | 0.47 | 0.79 |

**Table S5** Inheritance of the tribenuron-methyl resistance in the mutant line *K5* by fast screening method in green house

*ZS9*, *Zhongshuang No*.9; *K5*, the mutant line. χ^2^_0.05,1_=3.84, χ^2^_0.01,1_=6.63
