# Supplementary figures and images for: Inheritance and Molecular Characterization of a Novel Mutated AHAS Gene Responsible for the Resistance of AHAS-Inhibiting Herbicides in Rapeseed (Brassica napus L.)
Source: Int J Mol Sci. 2020 Feb 17;21(4):1345. doi: 10.3390/ijms21041345 (PMC7072869; doi:10.3390/ijms21041345)

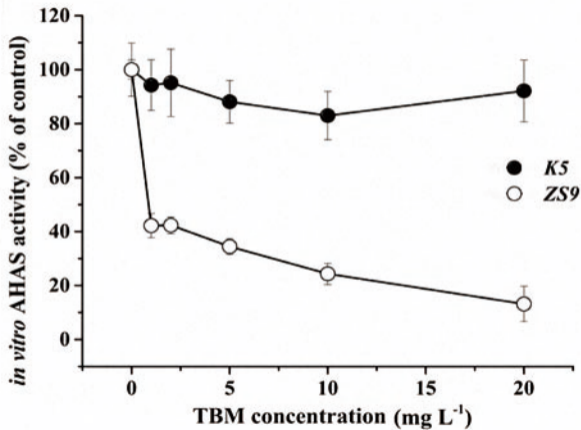

Supplement: Supplementary file 1 [file ijms-21-01345-s001.zip › Figure S2.pdf]

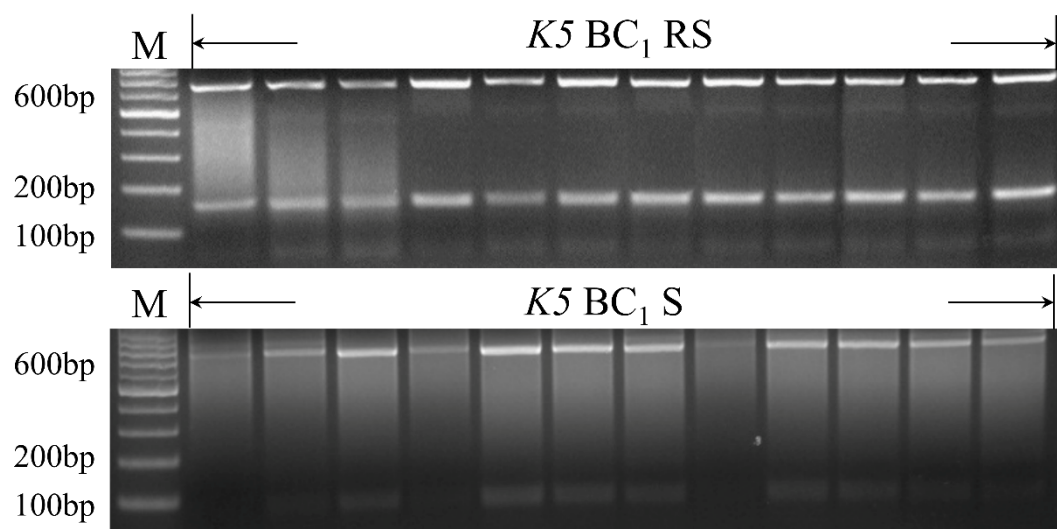

Supplement: Supplementary file 1 [file ijms-21-01345-s001.zip › Figure S3.pdf]

TBM 0.15 g a.i. ha<sup>-1</sup>

ZS9

K5 BC<sub>1</sub>

K5 F<sub>1</sub>

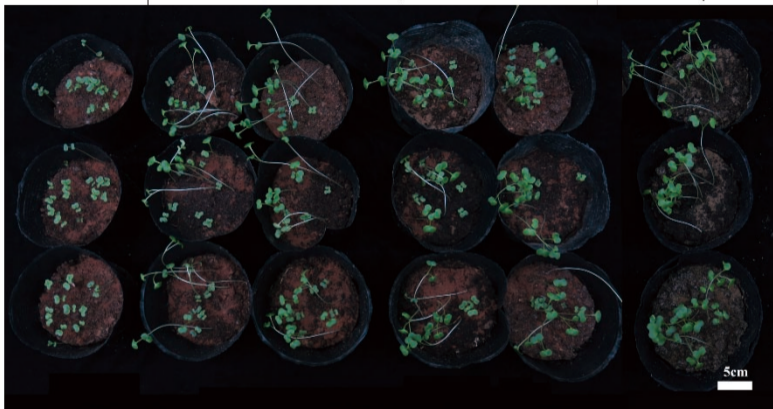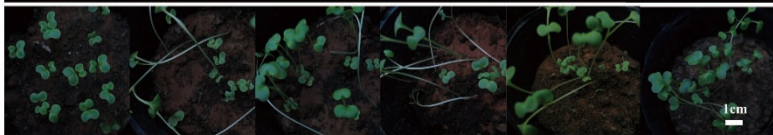

Supplement: Supplementary file 1 [file ijms-21-01345-s001.zip › Figure S4.pdf]
